# mSphere of Influence: Synthetic Biology of Natural Product Biosynthesis

**DOI:** 10.1128/mSphere.00954-19

**Published:** 2020-01-08

**Authors:** Mark C. Walker

**Affiliations:** a Department of Chemistry and Chemical Biology, University of New Mexico, Albuquerque, New Mexico, USA

**Keywords:** biosynthesis, metabolic engineering, natural product

## Abstract

Mark Walker studies the biosynthesis and engineering of bacterial natural products with the long-term goal of identifying new antibiotic compounds. In this mSphere of Influence, he reflects on how “Direct cloning and refactoring of a silent lipopeptide biosynthetic gene cluster yields the antibiotic taromycin A” by K. Yamanaka, K. A. Reynolds, R. D. Kersten, K. S. Ryan, et al. (Proc Natl Acad Sci USA 111:1957–1962, 2014, https://doi.org/10.1073/pnas.1319584111) impacted his thinking on using synthetic biology approaches to study natural product biosynthesis.

## Abstract (precis)

Mark Walker studies the biosynthesis and engineering of bacterial natural products with the long-term goal of identifying new antibiotic compounds. In this mSphere of Influence, he reflects on how “Direct cloning and refactoring of a silent lipopeptide biosynthetic gene cluster yields the antibiotic taromycin A” by K. Yamanaka, K. A. Reynolds, R. D. Kersten, K. S.

## COMMENTARY

Bacteria are fantastic chemists. Pharmaceuticals ranging from antibiotics to immunosuppressants either are, or are derived from, compounds produced by bacteria, or natural products. Even though the known bacterial natural products have been a tremendous benefit for society, the dramatic increase in available genome sequence data has revealed that these compounds are produced by a small fraction of the biosynthetic pathways that exist. Identifying the natural products produced by these pathways has the potential to aid in the identification of new lead compounds for pharmaceutical development, and in particular help address the growing threat of antibiotic-resistant bacterial infections. However, part of the reason the products of these pathways remain uncharacterized is that the pathways are often not transcribed, or silent, under laboratory conditions and are in slow-growing or genetically intractable organisms. One approach to overcome this hurdle is the heterologous expression of these pathways in well-characterized host organisms. However, this approach is often made challenging and labor-intensive due to the large size of the biosynthetic gene clusters encoding these pathways, which are often tens of thousands of base pairs long. During my postdoctoral training, I was inspired by an article from Bradley Moore’s lab, “Direct cloning and refactoring of a silent lipopeptide biosynthetic gene cluster yields the antibiotic taromycin A” (1). In this article, the Moore group utilizes transformation-associated recombination in yeast to directly clone biosynthetic gene clusters from genomic DNA, which facilitates their engineering and expression in heterologous hosts, thus laying out a route for more efficient identification of new natural products.

Transformation-associated recombination (TAR) cloning utilizes the homologous recombination capacity of yeast to insert linear DNA fragments into a vector backbone (2). To apply this technique to the cloning of natural-product biosynthetic clusters for expression in heterologous hosts, the Moore group designed a vector backbone with features for replication and selection in yeast and Escherichia coli, as well as features for chromosomal integration and selection in *Streptomyces* species. With this system they were able to directly clone a 73-kb DNA fragment harboring a silent biosynthetic gene cluster from the genomic DNA of the marine actinomycete *Saccharomonospora* sp. strain CNQ490, thereby achieving in one step what could have been months of work reconstructing the biosynthetic gene cluster from a cosmid library using more traditional techniques. Additionally, they were able to remove predicted transcriptional repressors in the biosynthetic gene cluster using yeast recombination-mediated PCR targeting (3). Upon transformation of the resulting vectors into the heterologous host, Streptomyces coelicolor, they were able to observe production of a new natural product that was similar to the antibiotic of last resort daptomycin but only with the vectors lacking the transcriptional repressors.

The design-build-test-learn cycle of synthetic biology (4) has the potential to allow us to gain a deeper understanding of how bacteria produce natural products in addition to discovering new ones. However, the build portion of this cycle, which needs to be repeated numerous times, taking months to complete raises real questions about the practicality of this approach. This article transformed my thinking about the feasibility of using synthetic biology to study natural-product biosynthesis. If we can more readily clone and alter biosynthetic gene clusters, we can begin asking questions about why heterologous expression fails and perform experiments to determine ways around it. With a deeper understanding of how heterologous hosts translate and transcribe these pathways, and therefore a more direct route to successful heterologous expression, we will be able to more efficiently work out the complex pathways that go into producing these compounds and discover new compounds that can benefit society.

The Moore group has followed up on this article, developing vectors that improve the efficiency of TAR cloning biosynthetic gene clusters (5) and allow for heterologous expression in Bacillus subtilis (6) and proteobacteria (7), facilitating the characterization of a number of new natural products.
